# Postural Abnormality as a Risk Marker for Leg Deep Venous Thrombosis in Parkinson’s Disease

**DOI:** 10.1371/journal.pone.0066984

**Published:** 2013-07-02

**Authors:** Kazushi Yamane, Fumiharu Kimura, Kiichi Unoda, Takafumi Hosokawa, Takahiko Hirose, Hiroki Tani, Yoshimitsu Doi, Simon Ishida, Hideto Nakajima, Toshiaki Hanafusa

**Affiliations:** Division of Neurology, the First Department of Internal Medicine, Osaka Medical College, Takatsuki, Japan; Oslo University Hospital, Norway

## Abstract

**Background:**

Pulmonary thromboembolism is a common cause of death in patients with autopsy-confirmed Parkinsonism. This study investigated the incidence of leg deep vein thrombosis in Parkinson’s disease and relationships between deep vein thrombosis and clinical/laboratory findings, including postural abnormalities as assessed by photographic measurements.

**Methods:**

This cross-sectional study assessed the presence of deep vein thrombosis using bilateral leg Doppler ultrasonography in 114 asymptomatic outpatients with Parkinson’s disease.

**Results:**

Deep vein thrombosis was detected in 23 patients (20%) with Parkinson’s disease. Deep vein thrombosis was located in the distal portion in 18 patients and in the proximal portion in 5 patients. No significant differences in age, sex, body mass index, disease duration, Hoehn-Yahr stage, anti-Parkinson’s drugs, or daily levodopa-equivalent dose were seen between deep vein thrombosis-positive and -negative groups. Univariate analysis for developing deep vein thrombosis in patients with Parkinson’s disease identified the following markers: long-term wheelchair use, bent knee, bent spine, and D-dimer elevation. Bending angles were significantly greater in the deep vein thrombosis-positive group at the knee and spine than in the deep vein thrombosis-negative group. Half of Parkinson’s disease patients with camptocormia had deep vein thrombosis. Among diabetes mellitus cases, long-term wheelchair use, bent knee over 15°, camptocormia, D-dimer elevation, the more risk markers were associated with a higher incidence of DVT. The presence of risk markers contributed to the development of deep vein thrombosis. On multivariate logistic regression analysis, a bent knee posture was strongly associated with an increased risk of deep vein thrombosis.

**Conclusion:**

Presence of leg deep vein thrombosis correlated with postural abnormalities in Parkinson’s disease. We recommend non-invasive ultrasonographic screening for leg deep vein thrombosis in these high-risk patients with Parkinson’s disease.

## Introduction

Sudden death occurs rarely in patients with Parkinson’s disease (PD). Among various causes of sudden death in patients with PD, pulmonary embolism (PE) is one potential cause [1]. In most patients with PE, thromboemboli originate in the deep veins of the lower extremities as deep vein thrombosis (DVT), which itself is not a dangerous condition. However, PE represents a serious and potentially fatal cardiovascular complication [2].

Multiple factors may be linked circumstantially to the development of DVT in PD, such as limited mobility [3], a long-term sitting position [4], and the parkinsonism-hyperpyrexia syndrome [5]. However, little information is available concerning DVT and PE in PD. One report described PE as the second most common cause of death in autopsy-confirmed PD cases [6]. Leg DVT theoretically poses a risk of progression into the larger veins of the leg, with the potential for subsequent PE. Since leg DVT is a main underlying cause of PE, examinations for the presence of leg DVT are invaluable in PD patients.

The aim of the present study was to assess the incidence of DVT in patients with PD and to identify risk markers including clinical (leg symptoms, past illness, use of supporting devices) and laboratory findings (D-dimer, etc.) for DVT in PD patients. Postural abnormalities such as a bent knee, a bent spine including camptocormia, and the Pisa sign (lateral bending) are important characteristics of PD [7]. This study focused particularly on correlations between postural abnormalities and DVT in PD. The combined effects of multiple risk markers on the development of DVT in PD patients were investigated.

## Patients and Methods

A total of 114 consecutive outpatients (58 men, 56 women) with a clinical diagnosis of PD were examined. The following patients were excluded from the study: receiving treatment with warfarin (n=5); had been in hospital within the previous 3 months (n=3); showed a reversible postural abnormality on administration of dopamine agonist (n=3); congestive heart failure (n=3); estimated glomerular filtration rate (eGFR) <30 mL/min (n=3); liver cirrhosis (n=2); thyroid disease with hypothyroid state (n=2); hypoalbuminemia (serum albumin <3 g/dl; n=1); or treated with anti-cancer drugs (n=1). PD was diagnosed based on the clinical criteria of the UK Parkinson’s Disease Society Brain Bank [8]. This cross-sectional study was performed at the time of follow-up visits during regular treatment with anti-PD drugs.

Ultrasonographic examinations of both legs were performed to screen for venous thrombosis in asymptomatic PD patients after obtaining informed consent from January to December in 2009. A color Doppler Prosound α-10 system (Hitachi-Aloka, Tokyo, Japan) with a 4- to 13-MHz linear transducer probe was used. The presence of DVT was evaluated with technical procedures using the milking reflex and the Valsalva maneuver, in addition to the compression method [9]. DVT was defined as the presence of a filling defect in more than one view or the presence of an abrupt cut-off in the venous column on ultrasonography, images of which were examined without knowledge of the clinical parameters. Thrombus characteristics such as proximal (femoral or supra-popliteal vein) or distal (infrapopliteal vein, gastrocnemius vein, soleus vein, peroneal vein, anterior tibial vein, or posterior tibial vein) location were evaluated.

Demographic and clinical data were collected, including age, sex, body mass index (BMI), disease duration from onset (years), Hoehn-Yahr (H–Y) stage, use of supporting devices (use of wheelchair over 4 hours per day, cane with walking, corset), clinical leg signs (pitting edema, superficial venous swelling, calf muscle cramps, restless leg), daily dose of anti-PD drugs (levodopa, cabergoline, pergolide, pramipexole, ropinirole, amantadine, selegiline, and daily levodopa-equivalent dose (LED) [10].

Laboratory findings that were collected included serum B-type natriuretic peptide (BNP), D-dimer, and glycated hemoglobin value (HbA1c). Individual estimated glomerular filtration rates (eGFRs) were calculated using the Modification of Diet in Renal Disease 4 formula [11]. Information about diabetes mellitus (DM), thyroid disease, and histories of cancer and bone fracture was obtained from history-taking for each patient. Moderate chronic kidney disease (CKD) was defined as an eGFR of 30-60 mL/minute/1.73 m^2^.

**Figure 1:**
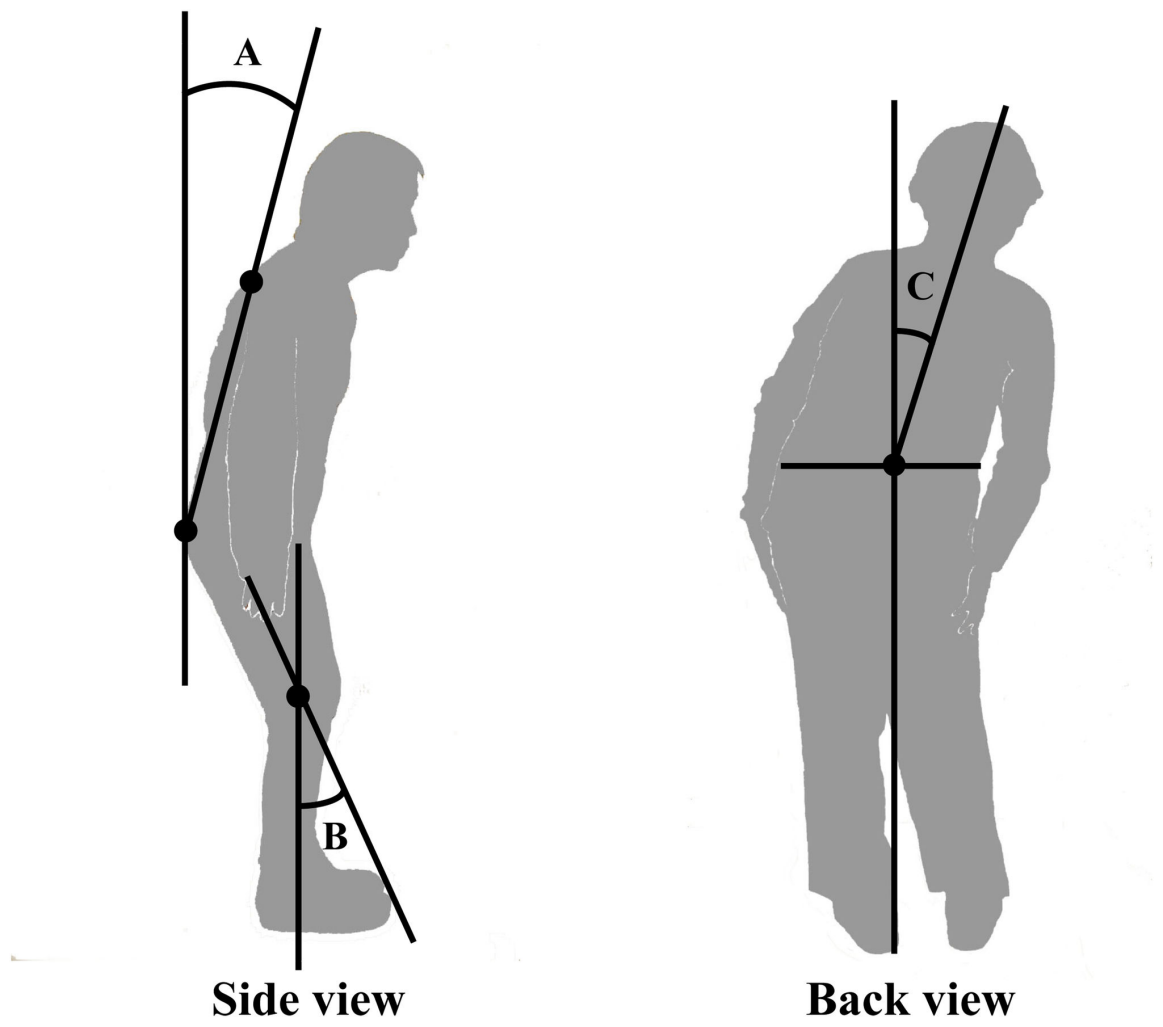
Measurements of postural abnormality in PD. Evaluations of bent spine and knee and the Pisa sign (lateral bending) are determined by the angle of the intersection of the basic axis and the movement axis. (A) Assessment of bent spine from a lateral view: (basic axis), a perpendicular line on the ground goes along the rear portion of the 5th lumbar vertebral body (movement axis), and a rear line links the 1st thoracic vertebral body to the 5th lumbar vertebral body. (B) Assessment of bent knee from the lateral view: (basic axis). The line passes along the center of the femoral bone (movement axis) and a median line links the lateral malleolus of the leg to the knee. (C) Assessment of the Pisa sign from the back view: (basic axis), a perpendicular line on the ground passes through the midpoint of the Jacoby line (movement axis) and a line links the 1st thoracic vertebral body to the midpoint of the Jacoby line.

Pitting edema was defined as which was evident on palpation and inspection of the anterior tibia after pressure for over 10 seconds. The presence of calf muscle cramps and restless legs was defined as the presence of these symptoms more than once a week. Simultaneously, postural abnormalities of the spine and knees and the Pisa sign (lateral bending of the trunk) were determined as the angle at the intersection of the basic axis and the movement axis using photographs from lateral and back views in a standing position (Figure 1). Camptocormia was diagnosed if the patient exhibited marked forward flexion (>45°) of the thoraco-lumbar spine in the standing posture.

For the present study, the combined effects of selected risk markers, such as the presence of DM, D-dimer over normal limits, camptocormia, wheelchair use over 4 hours per day, and bent knee (>15°), on the development of DVT were examined.

The protocol for this research was approved by the ethics committee at Osaka Medical College. Written informed consent was obtained from each patient prior to enrolment in this study. Data were analyzed using StatMate version 3.0 software (Microsoft Excel, Microsoft, Redmond, WA, USA). Differences between groups were analyzed using the χ^2^ test or Student’s *t*-test (two-tailed) for univariate analysis and logistic regression analysis for multivariate analysis. Since the postural angles were not normally distributed, non-parametric analyses were performed using the Mann–Whitney U-test.

## Results

### 1) The incidence of DVT in PD

A total of 114 patients who underwent extensive ultrasonography of bilateral lower extremities were enrolled. DVT was detected in 23 PD patients (20.1%). DVT in patients with PD was located distally in 18 patients and proximally in 5 patients. DVT was located in the left leg in 12 patients, the right leg in 4 patients, and bilaterally in 7 patients.

### 2)Clinical profiles and laboratory findings

The clinical profiles of DVT-positive (n=23) and DVT-negative groups (n=91) of PD patients were compared (Table 1). No significant differences were identified between groups for mean age, sex, BMI, duration of disease, or H–Y stage. Laboratory tests showed no differences in BNP, eGFR, or HbA1c at the time of evaluation between the groups. D-dimer levels were significantly higher in the DVT-positive group than in the DVT-negative group. The number of patients with above-normal D-dimer levels (>2 µg/dL) was also higher in the DVT-positive group than in the DVT-negative group (*p*<0.001). All patients with DVT presenting in both legs (n=7) showed above-normal D-dimer levels.

**Table 1:** (a) Overall clinical findings in the DVT-positive and -negative PD patient groups.

	**DVT (+)**	**DVT (-)**	***p***
**Case No.**	**23 (20.1%)**	**91**	
**age**	**73.3 ± 6.9**	**73.6 ± 9.4**	**0.86**
**Gender (M/F)**	**8/15**	**51/40**	**0.07**
**BMI**	**21.5 ± 2.8**	**21.8 ± 3.2**	**0.98**
**duration**	**5.69 ± 4.2**	**4.75 ± 4.0**	**0.33**
**H-Yahr stage**			
**Ⅰ-Ⅱ**	**8 (15%)**	**44**	**1.00**
**Ⅲ**	**9 (21%)**	**33**	**0.45**
**Ⅳ**	**6 (30%)**	**14**	**0.16**
**LED (mg/day)**	**336 ± 264**	**323 ± 314**	**0.62**
**BNP (pg/ml)**	**45.2 ± 30.8**	**46.4 ± 43.7**	**0.88**
**eGFR**	**64.7 ± 15.1**	**69.6 ± 17.6**	**0.18**
**HbA1c (%)**	**5.3 ± 1.3**	**5.3 ± 0.4**	**0.91**
**D-dimer (μg/dl)**	**2.7 ± 2.4**	**1.2 ± 2.1**	**0.01**
**> 2µg/dl**	**13 (57%)**	**5 (5%)**	**< 0.01**
**Diabetes Mellitus**	**5 (21%)**	**9 (10%)**	**0.12**
**Thyroid disease**	**1 (4%)**	**4 (4%)**	**0.99**
**history of cancer**	**1 (4%)**	**2 (2%)**	**0.57**
**history of fracture**	**4 (17%)**	**8 (9%)**	**0.23**
**CKD(eGFR<60)**	**9 (39%)**	**21 (23%)**	**0.12**
**Edema**	**12 (52%)**	**43 (47%)**	**0.67**
**Venous swelling**	**1 (4%)**	**4 (4%)**	**0.81**
**Calf muscle cramp**	**3 (11%)**	**27 (30%)**	**0.10**
**Restless leg**	**3 (13%)**	**8 (9%)**	**0.10**
**Stick**	**14 (23%)**	**46 (51%)**	**0.38**
**Wheelchair**	**10 (43%)**	**18 (20%)**	**0.02**
**Corset**	**3 (13%)**	**19 (21%)**	**0.39**
**Bent knee** (°)	**14.9**	**3.9**	**< 0.01**
**Bent spine** (°)	**19.8**	**8.0**	**< 0.01**
**Bent lateral** (°)	**3.8**	**2.8**	**0.27**
**Camptocornia (>**45°**)**	**4 (17%)**	**4 (4%)**	**0.03**

BMI, body mass indexLED, levodopa-equivalent dose per dayBent spine and knee and the Pisa sign are each shown as the average angle.Numbers I-IV represent case numbers in each Hoehn-Yahr stage as a standard of I+II

### 3) Complications

The complication of thyroid disease, DM, history of cancer and bone fracture, and moderate CKD were examined in both groups. The frequency of DM was relatively higher in the DVT-positive group than in the DVT-negative group, but the difference was not significant. No complications, including moderate CKD, were associated with the development of DVT.

### 4) Clinical leg signs and use of supporting devices

No significant differences in clinical leg signs, such as leg pitting edema, superficial venous swelling, calf muscle cramps, and restless legs, were seen between the groups. No patients had leg pain or Homan’s sign (pain in the back of the calf or knee during foot dorsiflexion). DVT was more frequent in patients with long-term wheelchair use (*p*=0.018), but no significant differences in use of a cane or corset were found.

### 5) Postural abnormalities

Photographic measurements showed that bending angles of the knee and spine were significantly greater in the DVT-positive group than in the DVT-negative group. Furthermore, half of the patents with camptocormia had DVT.

### 6) Anti-PD drugs and other drug use

The incidences of DVT in groups treated with each anti-PD drug and groups without each drug were compared as shown in Table 2. Of all the patients, 27 patients (24%) who were in a de novo state without any anti-PD drugs showed a relatively low frequency of DVT (3 patients, 11%), but the difference was not significant (*p*=0.179). The incidences of DVT showed no correlation to treatment with each anti-PD drug. No specific drugs were associated with the presence of DVT. No significant difference in LED was seen between the DVT-positive and -negative groups. Administration of low-dose aspirin (81 mg/day), statins, or calcium antagonists was not associated with the development of DVT.

**Table 2:** Anti-Parkinson drugs and other drug use compared between the DVT-positive group and the DVT-negative group.

	**DVT (**+**)**		**DVT (-)**		***p***
**Case No.**	**23**		**91**		
**No drug**	**3**	**(13%)**	**24**	**(26%)**	**0.18**
**Trihexyphenidyl**	**3**	**(13%)**	**4**	**(4%)**	**0.12**
**Selegiline**	**6**	**(26%)**	**21**	**(23%)**	**0.76**
**Cabergoline**	**2**	**(9%)**	**8**	**(9%)**	**0.99**
**Amantadine**	**8**	**(35%)**	**23**	**(25%)**	**0.36**
**Pramipexole**	**5**	**(22%)**	**16**	**(18%)**	**0.65**
**Pergolide**	**1**	**(4%)**	**6**	**(7%)**	**0.69**
**Ropinirole**	**4**	**(17%)**	**10**	**(11%)**	**0.40**
**Levodopa**	**17**	**(74%)**	**62**	**(68%)**	**0.59**
**Statin**	**2**	**(9%)**	**11**	**(12%)**	**0.64**
**Low dose aspirin**	**3**	**(13%)**	**20**	**(22%)**	**0.34**
**Calcium antagonist**	**7**	**(30%)**	**30**	**(33%)**	**0.82**

### 7) The combined effects of multiple risk markers on the development of DVT

**Figure 2:**
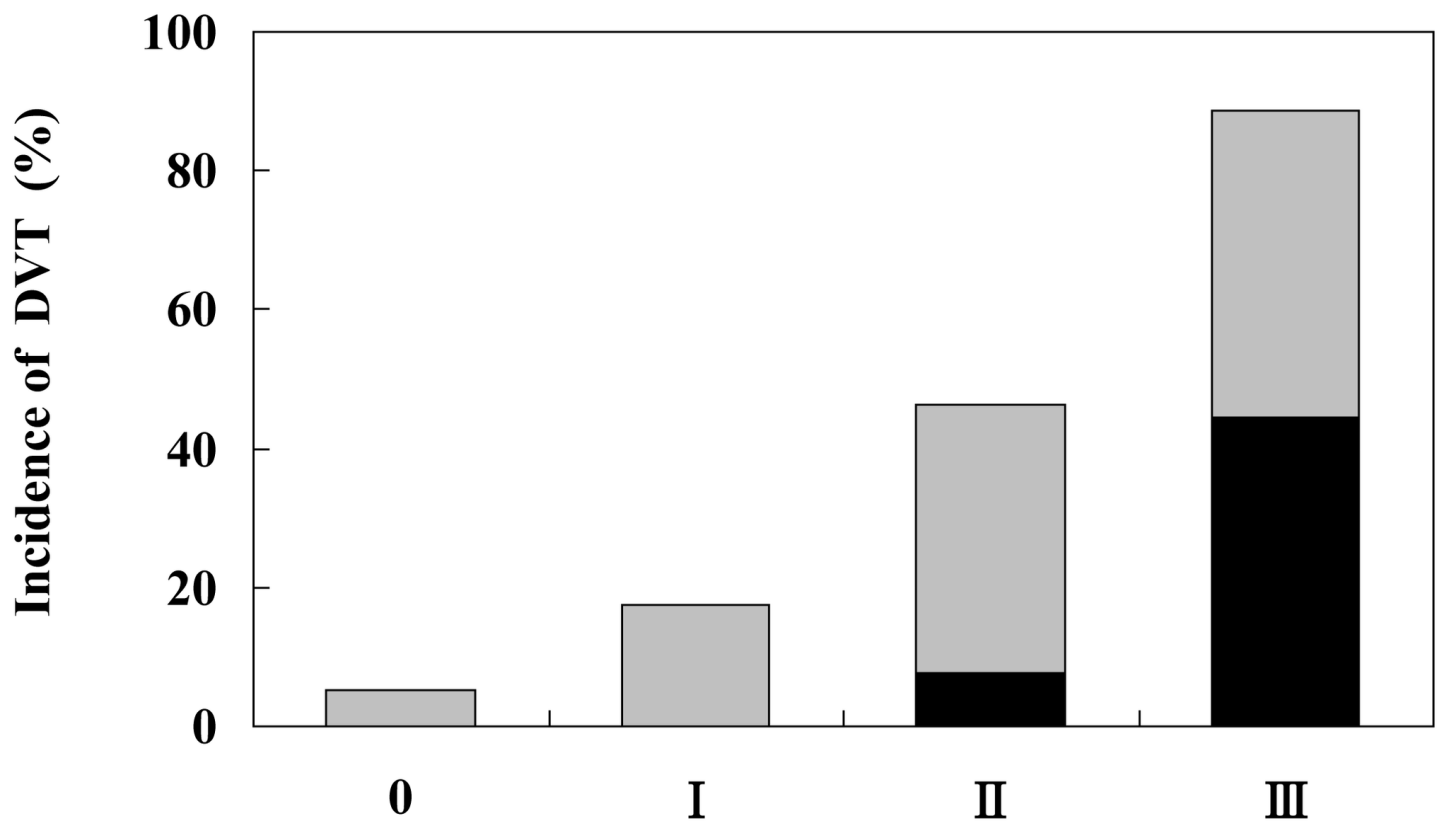
The correlation between the incidence of DVT and additional risk markers, including DM, D-dimer over normal limits, camptocormia, wheelchair use over 4 hours per day, and bent knee (>15°). 0 = no risk marker group (n=58) I = one risk marker group (n=34), II = two risk marker group (n=13), III = three or more risk marker group (n=9) (0 vs. I p=0.045 odds ratio 3.93, I vs. II p=0.045 odds ratio 4.0 II vs. III p=0.041, odds ratio 9.3). *With respect to the additional effect of these 5 risk* markers, a higher incidence of DVT is observed in patients having more risk markers. The vertical bar represents the % incidence of DVT in each risk marker group. The black box shows the % incidence of proximal DVT, and the grey one shows the % incidence of distal DVT.

As shown in Figure 2, the incidence of DVT was gradually increased for each additional risk-factor group (III, 89%; II, 46%; I, 18%; 0, 5%). This represented the additional effect of multiple risk markers. No risk marker was associated with a lower incidence (5%) to that of PD patients overall (20%) and the presence of two or more risk markers (groups II+III) was associated with a markedly higher incidence (42%, *p*<0.001). From the results of this survey, it can be seen that the development of DVT in PD involves a cluster of multiple risk markers. Importantly, 4 of 5 patients with proximal DVT belonged to the III risk-marker group.

### 8) Multivariate logistic regression analysis

Table 3 shows the results of the multivariate logistic regression analysis. These results indicated that the only significant independent variable related to DVT was the presence of a bent-knee posture.

**Table 3:** Multiple logistic regression analysis in relation to DVT.

	**Odds ratio**	**95% CI**	***P* value**
**Age**	**0.99**	**0.93-1.07**	**0.69**
**Gender (F/M)**	**2.40**	**0.68-8.56**	**0.18**
**Wheelchair**	**1.59**	**0.39-6.40**	**0.52**
**Bent spine**	**1.30**	**0.93-1.02**	**0.37**
**Bent knee**	**1.21**	**1.10-1.33**	**< 0.01**
**Pisa sign**	**0.97**	**0.83-1.11**	**0.58**
**Yahr stage**	**1.86**	**0.82-4.28**	**0.15**

## Discussion

The annual incidence of DVT is approximately 0.1% in the general population, 1.3% among hospitalized patients, and 1.8% among elderly patients between 65 and 69 years old [12,13]. In leg DVT associated with neurological disease, patients with acute illnesses, such as Guillain–Barré syndrome [14], relapsed MS [15], or stroke, appeared to show a high risk of DVT [16,17]. However, little information is available regarding DVT in chronic neurological illnesses such as PD. In 1999, Burbridge et al. [18] reported leg DVT in 4 of 81 PD patients (4.9%) (H–Y stage, 2.44±0.77; age, 70±8.3 years). In the present study, a higher incidence (20%) of DVT was seen in PD patients. The present study enrolled cases with relatively advanced PD (mean H–Y stage, 2.86±0.79) and older age (73.5±8.9 years). Technical progress in imaging and advanced resolution with ultrasonographic examination procedures has enabled more reliable detection of DVT when performed by experienced staff.

Distal DVT is often asymptomatic and found only incidentally. DVT usually starts anywhere in the veins and extends from the distal to the proximal veins in some cases [19,20], which supports the notion of distal DVT as a risk factor for PE [21]. This survey categorized patients with either distal or proximal DVT as “DVT-positive”.

Among clinical symptoms, leg pitting edema is frequently observed in patients with PD as a non-motor symptom [22]. No relationship was found between DVT and edema in the present study. Leg edema in PD may be associated with multiple factors, such as DVT, anti-PD drugs [23], autonomic failure, and latent cardiac, hepatic, or renal dysfunction. A direct correlation between leg edema and DVT can be masked by these factors related to edema. Calf muscle cramps were documented in 26% of PD cases in the present survey, but the presence of calf muscle cramps was not related to the presence of DVT in PD.

D-dimer is the most commonly used biological marker for assessing all kinds of systemic venous thromboembolism (VTE) [24]. In this study, the average D-dimer level was significantly higher in the DVT-positive group than in the DVT-negative group. However, only 57% of patients with DVT had a D-dimer level higher than the reference threshold value, probably due to the chronic nature of DVT development.

Venous stasis from prolonged sitting in a wheelchair combined with disuse muscle atrophy may enhance DVT formation and subsequent development of PE in a manner similar to “Economy Class Syndrome” [25]. Venous pressure is associated with height from the heart to the calf vein, and pressure rises while in the sitting position compared to the supine position [15]. We had previously proposed the term “wheelchair economy class syndrome” [4] for long-term wheelchair users with amyotrophic lateral sclerosis. We also have warned about the avoidable risk faced by long-term wheelchair users, including PD patients.

A significant association between the presence of DVT and postural abnormality in PD was found. This is the first report to find postural abnormality as a hallmark for the possibility of DVT. Postural abnormalities such as bent knee and spine are commonly associated with advanced PD [26]. Bent spine and knee show close relationships to each other. Posture with a bent spine increases stability by lowering the center of gravity, while flexion of the knee moves the center of gravity toward the toes to prevent backward falls. A bent-knee posture can induce directly venous congestion in the legs. Popliteal vein entrapment by the knee in a flexed position with prolonged sitting in a wheelchair may be closely associated with venous congestion in the leg and DVT development [27].

The comparisons across clusters of risk markers indicated that PD patients were more likely to develop DVT (DM, increased D-dimer over normal limits, camptocormia, wheelchair use over 4 hours per day, bent knee over 15°). These data suggest that the present criteria may provide additional information for the assessment of DVT risk in patients with PD. The previous report described that the risks of DVT are higher in diabetic patients than in the background population [28]. Several possible mechanisms might explain the association and additional effects between risk markers and DVT, such as endothelial dysfunction in DM, exaggeration of venous congestion with long-term wheelchair use, reduction of venous return by postural abnormality, and D-dimer levels indicating a hypercoagulable state [29].

Mechanical and pharmacological prophylaxis appears to be effective in preventing DVT and pulmonary embolism [30]. Although there is the fear that pharmacological prophylaxis increases bleeding in PD patients having postural instability, anticoagulation therapy had been performed in all PD patients with proximal DVT. In patients with distal DVT, mechanical VTE prophylaxis by elastic compression stockings had been in routine use. D-dimer levels dropped in a PD patient with distal DVT immediately after use of elastic compression stockings.

On multivariate logistic regression analysis, only a bent-knee posture was associated with an increased risk of DVT. In the future, outcomes related to the development of DVT from the distal to the proximal portion and resulting PE will be examined. Large randomized, prospective, clinical studies are needed to provide evidence to define the optimal VTE prophylaxis.

In conclusion, this study provides evidence of a higher risk for DVT in PD patients with a postural abnormality. We recommend ultrasonographic screening for DVT in PD patients with these high-risk markers.
